# Mining Potential Therapeutic Targets for T Cell Exhaustion in Osteoarthritis by Integrating Mendelian Randomization and Single‐Cell Sequencing

**DOI:** 10.1096/fj.202503295R

**Published:** 2026-01-28

**Authors:** Jiahong Li, Yi Ye, Yan Zhang, Wei Fang, Fei Lan, Qian Yang, Lun Wan, Jiang Hu, Chengwei Xiao, Kun Zhang

**Affiliations:** ^1^ Orthopaedic Department Sichuan Academy of Medical Sciences and Sichuan Provincial People's Hospital Sichuan China

**Keywords:** biomarkers, Mendelian randomization, osteoarthritis, single‐cell RNA sequencing, T cell exhaustion

## Abstract

Studies have shown that T cell exhaustion (TEX) indirectly influences the onset and progression of osteoarthritis (OA). This study aimed to ascertain biomarkers associated with TEX‐related genes (TEXRGs) in OA, offering potential targets for therapy and prognosis. OA related datasets were obtained from public databases. Candidate genes were ascertained by intersecting differentially expressed genes (DEGs) with TEXRGs. The expression quantitative trait locus (eQTL) data were then used as instrumental variables, and genes causally associated with OA were screened through Mendelian randomization (MR) analysis. Gene expression and receiver operating characteristic (ROC) analyses were carried out to identify biomarkers. Finally, functional enrichment, immune infiltration, drug prediction, molecular docking, and single‐cell analyses were conducted to explore the underlying biological mechanisms of OA. GPR65, LDLR, PLIN2, and TRIM14 were ascertained as biomarkers for OA. MR analysis revealed that PLIN2 acted as a protective factor, whereas GPR65, LDLR, and TRIM14 were risk factors of OA. LDLR and PLIN2 exhibited significantly lower expression in OA samples, whereas GPR65 and TRIM14 showed significantly higher expression. These four biomarkers were notably co‐enriched in the “lysosome” pathway. Six differential immune cell types were identified. Puromycin and gossypol were predicted as potential OA treatments. Finally, single‐cell analysis highlighted T cells and mast cells as key cell types in OA, with dynamic expression of GPR65 and PLIN2 observed throughout their differentiation. This study identifies GPR65, LDLR, PLIN2, and TRIM14 as biomarkers for OA, offering valuable insights that could support the development of targeted therapies.

## Graphical abstract

This study conducted an integrated analysis using multi‐source data, with specific data sources including the GEO database (encompassing datasets GSE55235, GSE55457, and GSE152805), literature‐mined gene sets, and the IEU OpenGWAS database (dataset ebi‐a‐GCST005814). First, candidate genes were initially screened via intersection analysis of differentially expressed genes (DEGs) from the GSE55235 dataset and literature‐derived TEXRGs; subsequently, functional annotation and interaction relationship characterization of these candidate genes were performed using GO functional enrichment analysis, KEGG pathway enrichment analysis, and protein–protein interaction (PPI) network analysis. On this basis, METTL1, ALDH18A1, and HNRNPH1 were finally identified as core key genes through integration with Mendelian randomization (MR) analysis, receiver operating characteristic (ROC) curve validation, and gene expression level verification. To systematically elucidate the biological significance of the key genes, further analyses were conducted: nomogram construction, chromosomal localization analysis, gene set enrichment analysis (GSEA), immune infiltration analysis, molecular regulatory mechanism characterization, potential therapeutic drug prediction, and molecular docking validation. Finally, combined with single‐cell transcriptome data, the expression characteristics of the key genes at the single‐cell level were analyzed.

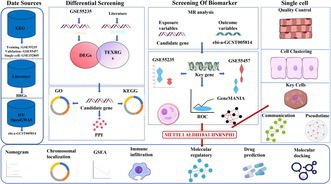

## 1 Introduction

Osteoarthritis (OA) is a chronic degenerative joint disease characterized by progressive degradation of articular cartilage, subchondral bone sclerosis, synovial inflammation, and osteophyte formation [1]. Pain is the main reason OA patients seek medical care, accompanied by joint stiffness, reduced movement, and functional impairment [2, 3]. The development and progression of OA involve multiple pathogenic factors, including joint trauma, mechanical loading, smoking, alcohol consumption, obesity, hormone use, genetic predisposition, and environmental influences [4, 5]. Currently, the global number of people affected by OA exceeds 200 million, with the incidence rate in women being approximately twice that in men, and it is projected that OA cases will increase by 40%–90% by 2025 [6]. According to statistics, musculoskeletal disorders, including OA, are among the leading causes of disability worldwide [7]. With the increasing number of individuals disabled by OA, the expected increase in healthcare burden represents a significant societal challenge [8]. Although nonsteroidal anti‐inflammatory drugs used in clinical practice can alleviate disease symptoms, they are unable to effectively delay or reverse the pathological progression of OA [9]. In cases of advanced OA, joint replacement surgery is frequently required to address pain and functional limitations. However, surgical risks and high costs limit its accessibility for all populations [10]. In terms of OA biomarker research, current studies are still limited by disease stratification, unclear sources of biomarkers, and poorly understood mechanisms [8, 11]. Consequently, advancing our understanding of OA molecular mechanisms and discovering more effective biomarkers are of critical importance for improving clinical outcomes.

Inflammation plays a pivotal role in modulating the initiation and persistence of pain associated with OA [12]. Substantial evidence has demonstrated a significant correlation between the severity of synovial inflammation and the clinical symptoms experienced by patients [13, 14]. OA is pathologically defined by chronic low‐grade inflammation and the infiltration of pro‐inflammatory factors in the synovium [15]. In the development of OA biomarkers, immune‐related phenotypes have consistently been one of the core focuses of diagnostic research, with a wealth of accumulated studies in this field. For instance, existing research has confirmed that autophagy‐related immune signature molecules (e.g., HSPA5, HSP90AA1, ITPKB) are associated with OA diagnosis [16]; other studies have explored aging‐related immune phenotypes, providing novel potential targets for OA diagnosis [17]; furthermore, additional research has successfully identified immune‐regulatory biomarkers related to OA diagnosis by integrating single‐cell sequencing data, which has further expanded the research scope in this field [18]. Chronic low‐grade inflammation has been identified as a contributing factor to TEX [19]. T cell exhaustion (TEX) refers to a pathological state in which T cells gradually exhibit diminished cytokine secretion, reduced proliferative capacity, and impaired cytotoxic activity following prolonged exposure to persistent antigenic stimulation [20]. TEX impairs the function of immune cells including CD8^+^ and CD4^+^ T cells [21]. Furthermore, in OA patients with hip implants, T‐cell exhaustion may be involved in regulating OA‐related tissue damage, forming a vicious cycle where inflammation leads to T‐cell exhaustion, which in turn results in uncontrolled inflammation; this cycle indirectly drives the progression of OA‐related pathological processes [22]. Therefore, exhausted T cells are unable to effectively regulate inflammatory responses or maintain balanced immune modulation, potentially contributing indirectly to the onset and progression of OA. Consequently, TEX and OA may share unexplored pathological mechanisms that warrant further investigation. However, most existing studies focus on single immune phenotypes or isolated molecules and lack systematic exploration of “TEX”—a critical immune state—in OA. Its regulatory mechanisms in OA, associations with disease progression, and potential as biomarkers for diagnosis/treatment have not been fully elucidated. This study aims to elucidate the mechanistic roles of TEX‐related genes in OA, thereby contributing to the development of novel therapeutic and prognostic approaches for OA patients.

Mendelian randomization (MR) is a genetic method that utilizes genetic variants as instrumental variables (IVs) to assess the potential causal relationship between the studied exposure and the target outcome [23]. Given that genes are randomly assigned during fertilization, MR functions as a natural randomization, helping to sidestep the confounding factors and reverse causation that often beset traditional studies [24]. Moreover, an additional benefit that MR confers is its capacity to probe into the impacts of infrequent genetic variants or exposures that are arduous to quantify on the results [25, 26]. In MR analysis, the expression of quantitative trait loci (eQTL) data is often used as IV. By simulating randomized controlled trials through random allocation of genetic variations at conception, the interference of confounding factors and reverse causal relationships on causal inference is significantly reduced [27] Within the domain of OA, MR has been employed to explore potential correlations between factors such as lifestyle [28], iron status [29], metabolites [30], and OA. Single‐cell RNA sequencing (scRNA‐seq) is a high‐throughput sequencing technique that analyzes the transcriptome at the individual cell level, revealing the expression profiles of all genes across the entire genome within a single cell [31].

Given that the specific mechanism of TEX in the occurrence and development of OA is not yet clear, this study innovatively integrates multidimensional analysis strategies to explore its molecular mechanism and clinical value in depth. The study used eQTL data as a genetic tool variable for TEXRGs, combined with MR analysis system to evaluate the potential causal effects of candidate genes on OA, screened key biomarkers driven by TEX in OA, and further explored the interaction mechanism between their regulatory network and immune microenvironment. At the same time, combined with single‐cell RNA sequencing technology, the dynamic cellular composition and molecular characteristics of joint tissues in OA patients were revealed. In summary, this study aims to provide novel molecular targets for the precise diagnosis of OA and lay a solid theoretical foundation for the development of personalized treatment strategies targeting the TEX pathway, providing theoretical support for clinical treatment.

## 2 Materials and Methods

### 2.1 Data Collection

In this study, datasets related to osteoarthritis (OA) were retrieved from the Gene Expression Omnibus (GEO) database (https://www.ncbi.nlm.nih.gov/geo/). Specifically, the GSE55235 dataset (GPL96 platform) included transcriptomic data from 10 OA and 10 normal synovial tissue samples, while the GSE55457 dataset (GPL96 platform) also comprised transcriptomic data from 10 OA and 10 normal synovial tissue samples. Additionally, the single‐cell RNA sequencing (scRNA‐seq) dataset GSE152805 (GPL20301 platform) was utilized, which contained data from three OA synovial tissue samples.

Mendelian randomization (MR) data for OA were mined from the IEU Open GWAS database (https://gwas.mrcieu.ac.uk/). The MR dataset for OA (ebi‐a‐GCST005814) included data from 50 508 Europeans (10 083 cases and 40 425 controls) and 15 845 511 single nucleotide polymorphisms (SNPs). Additionally, expression quantitative trait locus (eQTL) data on exposure factors were also retrieved from the IEU OpenGWAS database.

**FIGURE 1:**
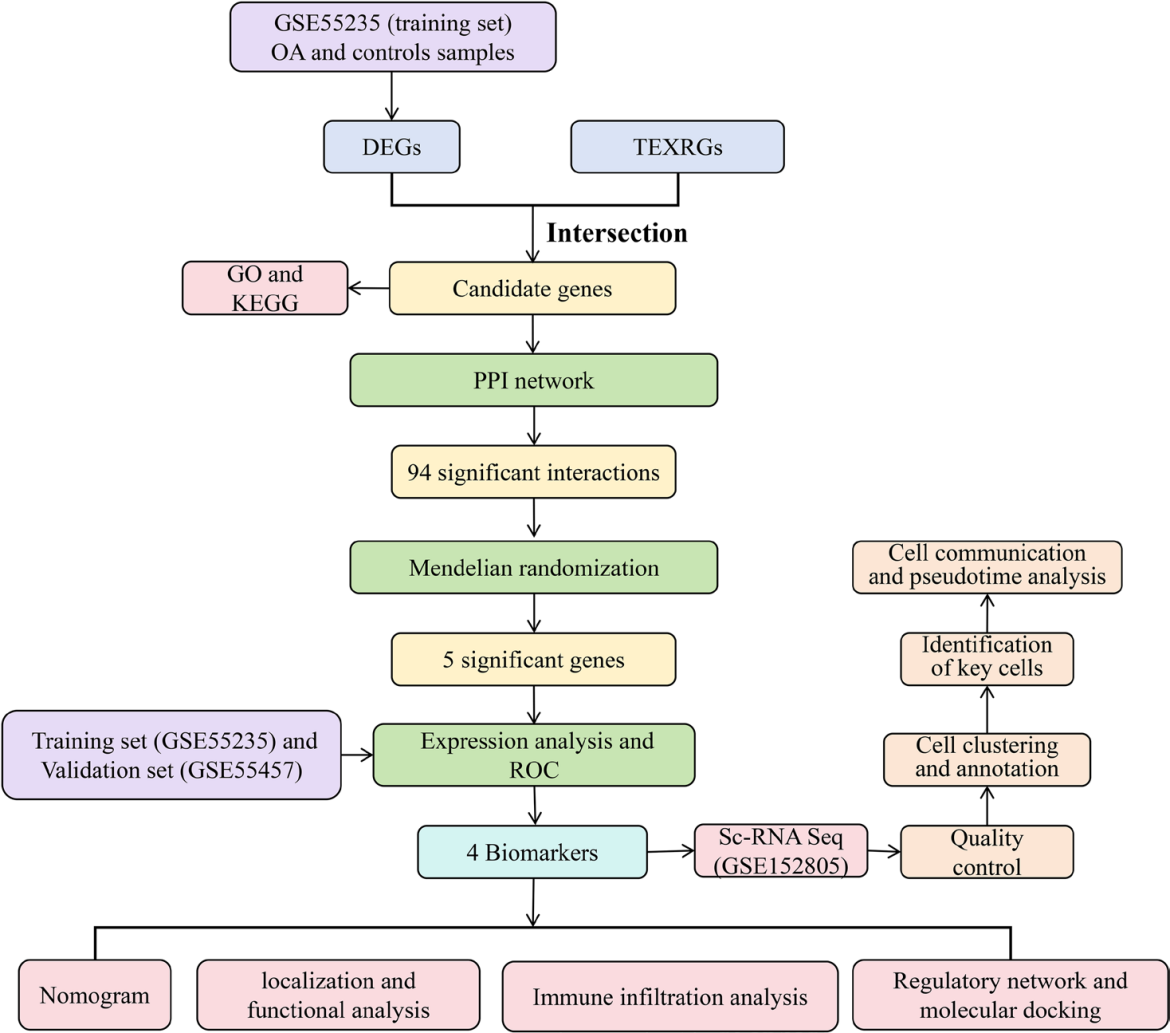
The flowchart of bioinformatics analysis in this study.

Moreover, 683 T cell exhaustion‐related genes (TEXRGs) were collected from the literature [32] (Table S1). The flowchart of this study was shown in Figure 1.

### 2.2 Differential Expression Analysis

Differentially expressed genes (DEGs) between OA and normal samples in the GSE55235 dataset were ascertained by “limma” package (v 3.54.0) [33] (adj. *p* < 0.05 and |log_2_ fold‐change (FC)| > 1) [34, 35]. The DEGs were visualized via a volcano plot and a heat map. The volcano plot was created with “ggplot2” (v 3.4.1) [36], while the heat map, displaying only the top 10 up‐ and down‐regulated DEGs ranked by |log_2_ FC| from high to low, was generated using “ComplexHeatmap” package (v 2.14.0) [37].

### 2.3 Identification and Function Analyses of Candidate Genes

The DEGs were then further intersected with the TEXRGs to identify candidate genes by “VennDiagram” package (v 1.7.3) [38]. Subsequently, Gene Ontology (GO) and Kyoto Encyclopedia of Genes and Genomes (KEGG) analyses were conducted utilizing the “clusterProfiler” package (v 4.7.1.3) [39] (adj. *p* < 0.05). Additionally, to examine protein interactions among the candidate genes, a protein–protein interaction (PPI) network was constructed using the STRING database (https://string‐db.org/) with a confidence score > 0.9. The PPI network was then visualized using Cytoscape software (v 3.7.2) [40].

### 2.4 Data Pre‐Processing for Mendelian Randomization (MR) Study

In the MR study, eQTLs of candidate genes were considered as exposure factors, with OA as the outcome. The classical MR analysis confirmed the satisfaction of three key assumptions throughout the analysis: (i) the independence assumption (instrumental variables [IVs] were not associated with any confounders); (ii) the association assumption (IVs directly affected the exposure); (iii) the exclusivity assumption (IVs could only influence the outcome variable through the exposure factors and not through other pathways).

Firstly, the “extract_instruments” function from the “TwoSampleMR” package (v 0.5.6) [41] was utilized to read data for exposure factors and filter IVs (*p* < 5 × 10^−8^). This criterion ensures a strong correlation between IVs and the exposure to avoid weak instrument bias, and also ensures that the selected IVs have detectable signals in the outcome (by referencing the significance threshold for outcome‐related SNPs), thereby preventing the inclusion of completely invalid IVs. Subsequently, SNPs in linkage disequilibrium (LD) were removed (clump = TRUE, *r* ^2^ < 0.001, kb < 10 000). This step was intended to avoid high correlation among multiple IVs, which could lead to distorted estimates in MR‐Egger or IVW analyses. Next, the “extract_outcome_data” function was employed to choose SNPs associated with the outcome under conditions of proxies = TRUE and *r* ^2^ = 0.8. SNPs associated with the outcome were then excluded, while those associated with the exposure factors were retained. The strength of each SNP was evaluated using the *F*‐statistic; SNPs with an *F*‐value < 10 were considered weak instrumental variables (IVs) and required exclusion or reporting of bias risk. Meanwhile, the MR‐RAPS method was employed to adjust the IVs. Lastly, the effect alleles and effect sizes were harmonized using the “harmonise_data” function for further analysis.

### 2.5 MR Study, Sensitivity Analysis, and Steiger Test

After screening IVs, five algorithms IVW [42], MR Egger [43], weighted median [44], simple mode [45], and weighted mode [44] were employed for MR study combining with the “mr” function. Among these methods, IVW was deemed the most critical. The screening criteria for the MR study were set at *p*_IVW_ < 0.05 and SNPs ≥ 3. Additionally, gene‐level effective‐median‐based Mendelian randomization for inferring the causal genes (EMIC) analysis was performed to further explore the causal relationships between exposure factors and outcomes. To visually assess the distribution of our random variables, conditional quantile–quantile plot (Q–Q plot) was plotted, which provided a graphical representation to verify whether the variables conformed to a known distribution. Following this, scatter plots were generated to assess the correlation between exposure factors and the outcome. Forest plots were created to visualize the diagnostic effectiveness of exposure factors on the outcome, while funnel plots were used to examine the symmetry of causal effects distribution.

Besides, a series of sensitivity analyses were executed to ensure the robustness of the MR results. These analyses comprised heterogeneity test conducted with “mr_heterogeneity” [46], with the criteria of *p* > 0.05 for Cochran's *Q* test and *I* ^2^ = 0; a *p* value > 0.05 indicates the absence of heterogeneity, and the fixed‐effects inverse variance weighted (IVW) method was thus used for subsequent analyses. Subsequently, assessment of horizontal pleiotropy using “mr_pleiotropy_test” [47] and MR‐PRESSO method with a criterion of *p* > 0.05; a *p* value > 0.05 suggests no horizontal pleiotropy, confirming the reliability of the results. Finally, leave‐one‐out (LOO) analysis employing “mr_leaveoneout” [48], which evaluates whether a single SNP drives the overall results: a substantial change in results following the removal of a specific SNP indicates that the outcome is sensitive to that instrument, whereas minimal changes in the effect of remaining SNPs on the outcome variable after the stepwise removal of each SNP demonstrate that the MR analysis results are reliable and stable. Subsequently, Steiger analysis was employed to determine the directionality of the causal relationship by “TwoSampleMR.” The criteria for passing the Steiger analysis were correct causal direction = TRUE and *p* < 0.05. Overall, after conducting these comprehensive analyses, significant genes were identified for further investigation. As the final step, the Global test was implemented to evaluate the overall statistical significance of the findings.

### 2.6 Identification of Biomarkers and Construction of Nomogram

Gene expression analyses of significant genes in the GSE55235 and GSE55457 datasets were performed separately using the Wilcoxon test (OA vs. normal). Genes that exhibited significant differences (*p* < 0.05) and consistent expression patterns across both datasets were selected as candidate biomarkers for further investigation. Receiver operating characteristic (ROC) curves were generated by the “pROC” package (v 1.18.0) [49] to evaluate the diagnostic potential of the candidate biomarkers for OA in both the GSE55235 and GSE55457 datasets. Biomarkers were selected based on an area under the curve (AUC) greater than 0.7.

Based on the selected biomarkers, a nomogram was built to predict the risk of OA by “rms” package (v 6.5.0) [50]. Subsequently, a calibration curve was plotted to assess the accuracy of the nomogram. The Hosmer–Lemeshow (HL) test was performed. If *p* > 0.05 and the slope was close to 1, it indicated that there was no significant difference between the predicted values and the true values, suggesting a better model fit and more accurate predictions.

### 2.7 Chromosomal Localization and Function Enrichment Analyses

To reveal the positions of the biomarkers in the genome and further infer their potential evolutionary conservation, the “RCircos” package (v 1.2.2) [51] was used to generate a distribution map of their chromosomal loci. Additionally, we carried out enrichment analysis of the biomarkers. Specifically, in the GSE55235 dataset, Spearman correlations were computed between the biomarkers and other genes, and these correlations were ranked from high to low. The background gene set for enrichment analysis was gained from the “c2.cp.kegg.v2023.1.Hs.symbols.gmt” file, which was downloaded from the Molecular Signatures Database (MSigDB, https://www.gsea‐msigdb.org/gsea/msigdb/). Gene Set Enrichment Analysis (GSEA) was then conducted to assess the enrichment of the ranked genes within this background set (*p* < 0.05 and |NES| > 1).

### 2.8 Immune Infiltration Analysis

To investigate immune cell infiltration in OA and normal samples, an immune infiltration analysis was conducted in the GSE55235 dataset. CIBERSORT algorithm (v 1.03) [52] was first used to calculate the infiltration proportions for 22 immune cell types [53]. Samples that had a *p* > 0.05 were excluded from further analysis. Wilcoxon test was then applied to compare infiltration proportions between the OA and normal samples, identifying immune cells with obvious differences (*p* < 0.05) for further investigation. Subsequently, Spearman correlation analysis was performed via “psych” package (v 0.92) (https://CRAN.R‐project.org/package=psych) to explore the relationships between differential immune cells, as well as between biomarkers and differential immune cells, with correlations considered significant at |cor| > 0.3 and *p* < 0.05.

### 2.9 Regulation Network Analysis

To explore the upstream regulatory mechanisms of biomarkers in osteoarthritis (OA) and their interactions during disease progression, the “multiMiR” package (v 1.18.0) [54] was employed to predict the miRNAs targeting biomarkers. Specifically, targetscan (http://www.targetscan.org) and miranda (http://www.microrna.org) databases were utilized to predict miRNAs targeting the biomarkers. The key miRNAs were ascertained by overlapping the predicted miRNAs from both databases. Then, the Starbase database (http://starbase.sysu.edu.cn/starbase2/index.php) was utilized to predict the miRNA–lncRNA relationship. Finally, the lncRNA–miRNA–mRNA network was constructed and visualized using Cytoscape software to systematically clarify the relationships among lncRNAs, miRNAs, and mRNAs, offering new insights into the roles of these biomarkers in OA development.

### 2.10 Drug Prediction and Molecular Docking

To explore potential drugs for OA treatment based on the identified biomarkers, the DGIdb database (https://www.dgidb.org/) was utilized. The top 30 drugs targeting each biomarker were selected to construct and visualize a biomarker–drug interaction network using Cytoscape. Following this, to evaluate the binding affinity of the drugs to the biomarkers, we focused on drugs with a *p*‐value < 0.05 and selected those with the highest correlation to the biomarkers for molecular docking studies. The 3D structures of the biomarkers (acting as receptors) were retrieved from the Uniprot database (https://www.uniprot.org/), while the 3D molecular structures of the drugs (acting as ligands) were mined from the PubChem database (https://pubchem.ncbi.nlm.nih.gov/). Molecular docking analysis was performed using CB‐Dock2 (https://cadd.labshare.cn/cb‐dock2/php/index.php), and the binding energy was calculated. A binding energy of less than −5 kcal/mol typically indicates a high affinity between the drug and the biomarker, suggesting effective molecular binding.

### 2.11 Single‐Cell Analysis

The scRNA‐seq data from GSE152805 were analyzed using the “Seurat” package (v 5.0.1) [33]. Initially, the retained cells met the following quality control (QC) thresholds: 200 ≤ *n*Feature‐RNA (genes per cell) ≤ 6000, 500 ≤ *n*Count‐RNA (total RNA count per cell) < 40 000, and percent.mt (proportion of mitochondrial gene expression) < 10%. After QC, the data were normalized using the NormalizeData function. The normalization method was as follows: for each cell, the expression value of each gene was divided by the total expression value of all genes in that cell; the resulting quotient was then multiplied by a scaling factor of 10 000, with 1 added to this product afterward, followed by a logarithmic transformation to generate the normalized data. Highly variable genes (HVGs) were ascertained utilizing the FindVariableFeatures function (with selection.method = “vst”), and the top 10 HVGs were labeled and visualized using the “LabelPoints” function. Principal component analysis (PCA) was then carried out on the HVGs using the ExPosition package (v 2.8.23) (https://CRAN.R‐project.org/package=ExPosition). Principal components (PCs) were selected based on the JackStraw function (*p* < 0.05). Dimensionality reduction was conducted using UMAP with a resolution of 0.8. The FindAllMarkers function was used to ascertain marker genes for each cell cluster in the single‐cell dataset, applying thresholds of logfc.threshold = 0.5, min.pct = 0.25, and return.thresh = 0.01. According to the screened marker genes, the different cell clusters obtained were annotated to known cell types by combining the cellMarker database (http://yikedaxue.slwshop.cn/) and the “singleR” package (v 2.0.0) [55]. Notably, the distribution of biomarkers across different cell types in the OA samples was visualized using UMAP plots from the scRNA‐seq dataset GSE152805. Cells exhibiting prominent expression of most biomarkers were identified as key cells.

### 2.12 Cell‐to‐Cell Communication Analysis and Pseudo‐Temporal Analysis

To gain a deeper understanding of the interactions between key cells and other annotated cell clusters in the GSE152805 dataset, cell‐to‐cell communication analysis was carried out using the CellChat package (v 1.6.1) [56]. Specifically, CellPhoneDB (https://www.cellphonedb.org/), a signaling molecule interaction database, was employed to analyze intercellular ligand–receptor interactions (*p* < 0.05 and log_2_mean [molecule 1, molecule 2] ≥ 0). Finally, to explore the differentiation states and trajectories of key cells, pseudo‐temporal analysis was performed. First, dimensionality reduction and clustering of the key cells were conducted, and the cells were annotated into different subtypes. Next, “Monocle2” package (v 2.22.0) [56] was used to perform pseudo‐temporal analysis on the subtype cells following re‐clustering and dimensionality reduction. Additionally, the expression changes of biomarkers throughout the pseudo‐temporal progression were analyzed using the DifferentialGeneTest function.

### 2.13 Statistical Analysis

R (v 4.2.3) was utilized to conduct statistical analysis. Difference analysis between two groups was executed via the Wilcoxon test (*p* < 0.05). Notably, **** represented *p* < 0.0001, *** represented *p* < 0.001, ** represented *p* < 0.01, * represented *p* < 0.05, and ns represented *p* > 0.05 (no significant differences).

## 3 Results

### 3.1 Acquisition and Function Analysis of Candidate Genes

**FIGURE 2:**
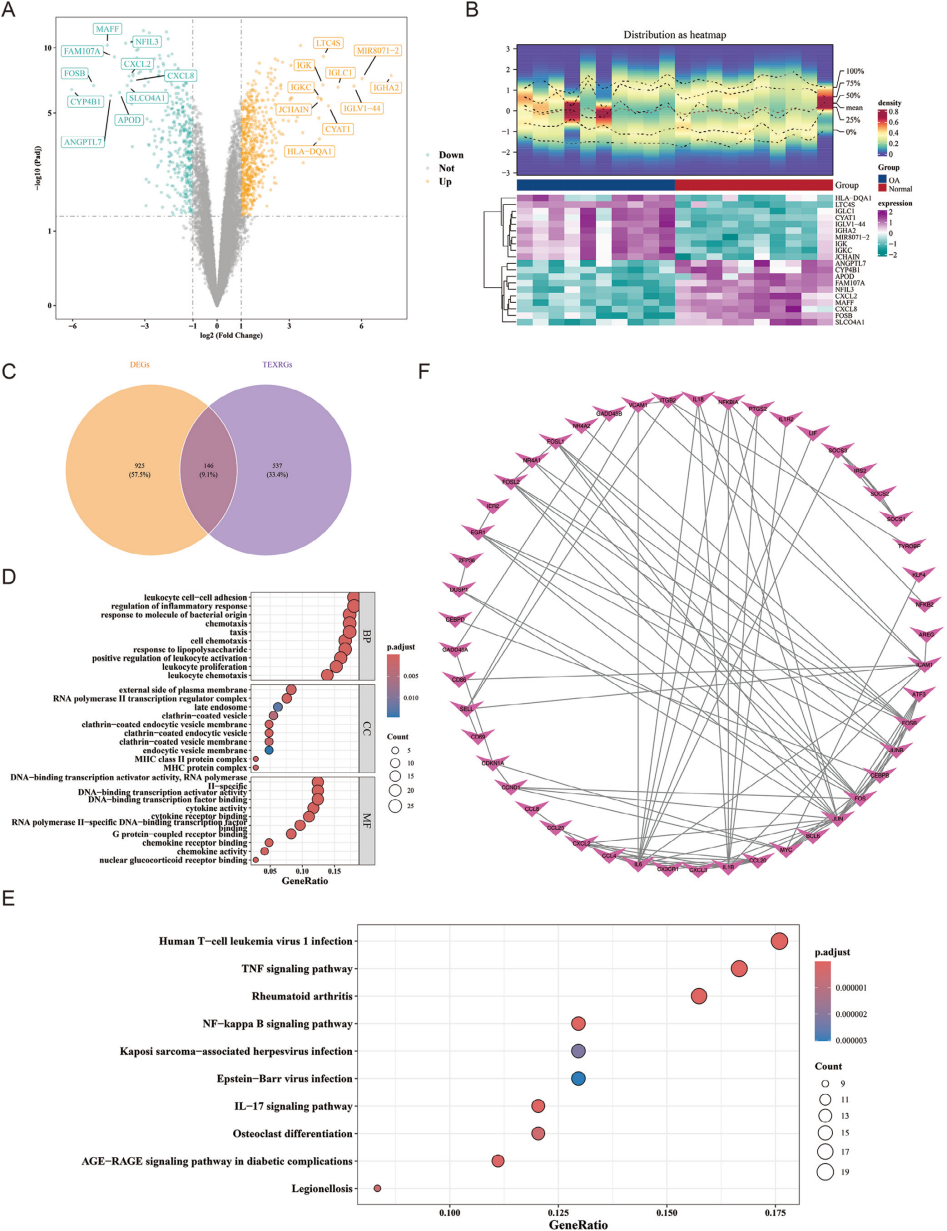
Acquisition and function analysis of candidate genes. (A) Volcanic diagram of DEGs. Yellow: Up‐regulated genes; Green: Down‐regulated genes; Gray: Insignificant gene. The horizontal axis represents the log_2_FC values of genes, while the vertical axis represents the −log_10_(adj. *p*) values of genes. (B) Heatmap of DEGs. The upper panel shows a density heatmap of gene expression. In the middle section, blue represents the osteoarthritis (OA) group; red represents the normal group. The lower panel displays an expression heatmap, where each column corresponds to a sample and each square represents a gene. (C) Venn diagram of candidate genes. Orange: DEGs; Purple: TEXRGs. The intersecting genes are the candidate genes. (D) Bubble chart displaying Gene Ontology (GO) enrichment results for candidate genes across three domains: Biological process (BP), cellular component (CC), and molecular function (MF). The vertical axis represents different GO entries, while the horizontal axis shows the gene ratio per entry. Colors range from blue to red, indicating enrichment significance. (F) KEGG enrichment analysis of candidate genes. The size of bubbles usually reflects the number of genes associated with a certain pathway. The larger the bubble, the more genes are involved in this pathway. The color of bubbles represents the level of significance, usually the redder the color, the smaller the *p*‐value, indicating a more significant enrichment of the pathway; The bluer the color, the lower the significance. (F) PPI network. Different nodes represent different candidate genes, and connecting lines indicate the interaction between two candidate genes.

In GSE55235 dataset, 1071 DEGs were ascertained, including 674 up‐ and 397 down‐regulated DEGs in OA samples (Figure 2A,B; Table S2). These 1071 DEGs were then overlapped with 683 TEXRGs, leading to the identification of 146 candidate genes (Figure 2C). Functional analysis revealed that the candidate genes were notably associated with 1107 GO terms. Notable associations included terms such as “leukocyte cell‐cell adhesion” (BP), “external side of plasma membrane” (CC), and “DNA‐binding transcription factor binding” (MF) (Figure 2D; Table S3). For KEGG pathways, the candidate genes were notably enriched in 201 pathways, for instance “Human T‐cell leukemia virus 1 infection” and “TNF signaling pathway” (Figure 2E; Table S4). Additionally, a PPI network was constructed after removing 97 outlier genes, revealing 94 significant interactions (Figure 2F). Notably, IL6 exhibited strong interactions with several genes, such as ICAM1 and IL18, within the network.

### 3.2 The Significant Genes Had Significant Causal Relationship With OA

Among the 146 candidate genes, MR analysis indicated that five genes exhibited significant causal relationships with OA at *p*_IVW_ < 0.05 and SNPs ≥ 3. The strength of IVs was fully evaluated by *F* statistics and MR‐RAPS (Table S4). Notably, LDLR (odds ratio [OR] = 1.1682, 95% CI = 1.0212–1.3363, *p* = 0.0235), GPR65 (OR = 1.0904, 95% CI = 1.0092–1.1780, *p* = 0.0283), and TRIM14 (OR = 1.1282, 95% CI = 1.0123–1.2573, *p* = 0.0292) were identified as risk factors for OA, while PLIN2 (OR = 0.9147, 95% CI = 0.8616–0.9711, *p* = 0.0035) and TGFBR3 (OR = 0.8245, 95% CI = 0.7044–0.9651, *p* = 0.016) showed protective effects (Table 1). Additionally, our analysis revealed notable findings regarding specific genes. Both TGFBR3 and PLIN2 exhibited negative effects in the IVW and EMIC methods, with their 95% CI not crossing the zero line (Figure S1A). Conversely, GPR65 and TRIM14 displayed clear positive effects in these methods, although their 95% CI had slight crossovers at zero (Figure S1A). For LDLR, the 95% CI in the IVW method did not cross zero, indicating a positive effect (Figure S1A). These results highlighted the consistency between the IVW and EMIC methods and suggested robust causal effects of these genes on the phenotype. Furthermore, the Q–Q plot for osteoarthritis (OA; ebi‐a‐GCST005814) showed a lambda genomic control (GC) of 1.01 (Figure S1B). This value indicated that the overall false positive risk was not significantly elevated. Only a few deviation points were observed at the upper right end of the plot, suggesting that these SNPs might indeed harbor genuine correlation signals.

**TABLE 1:** The results of MR.

Gene	SNP	IVW (*p*)	OR (95% CI)
PLIN2	6	0.0035	0.9147 (0.8616–0.97112)
TGFBR3	4	0.016	0.8245 (0.7044–0.9651)
LDLR	7	0.0235	1.1682 (1.0212–1.3363)
GPR65	4	0.0283	1.0904 (1.0092–1.1780)
TRIM14	3	0.0292	1.1282 (1.0123–1.2573)

*Note:* In the table, “Gene” refers to the gene name; “SNP” denotes the number of single nucleotide polymorphism sites; “IVW (*p*)” denotes the inverse variance‐weighted *p*‐value, used to assess the statistical significance of the association between the gene and the exposure factor or outcome; “OR (95% CI)” represents the odds ratio and its 95% confidence interval, reflecting the degree of influence of the gene on the outcome and its confidence range.

Scatter plots were used to visually represent these findings, illustrating a negative slope for PLIN2 and TGFBR3, indicating protective associations, while LDLR, GPR65, and TRIM14 showed positive slopes, indicating risk associations (Figure S2). Forest plot results showed that the MR effect size of LDLR, GPR65, and TRIM14 was greater than 0, while the effect size of PLIN2 and TGFBR3 was less than 0 in the IVW method (Figure S3). The randomness test indicated that IV was evenly distributed along both sides of the IVW line, suggesting that the MR analysis adhered to Mendel's second law of random assortment (Figure S4). Furthermore, there was no evidence of heterogeneity (*p* > 0.05) (Table 2), and no confounding factors were identified in the MR study (*p* > 0.05) (Table 3). Additionally, LOO analysis revealed no outliers, indicating the reliability of the MR results (Figures S3 and S5). Besides, the results of MR‐PRESSO were displayed in Table S5. It should be noted that there were no results available for TRIM14. The Steiger test confirmed the causality of the five genes with OA was genuine and unaffected by reverse causality (Table 4). Finally, the results of the Global test were presented in Table S6. It should be noted that there were no results available for TRIM14.

**TABLE 2:** The results of heterogeneity test.

Gene	Method	*Q*	*Q*_df	*Q*_*p*val
GPR65	MR Egger	1.597746469	2	0.449835538
	Inverse variance weighted	1.610847806	3	0.656932661
LDLR	MR Egger	6.887922543	5	0.229109993
	Inverse variance weighted	8.425181887	6	0.208578181
PLIN2	MR Egger	3.166842909	4	0.530302973
	Inverse variance weighted	3.783443821	5	0.580996704
TGFBR3	MR Egger	0.194305034	2	0.907417599
	Inverse variance weighted	0.548713819	3	0.908066015
TRIM14	MR Egger	0.047327278	1	0.827781043
	Inverse variance weighted	0.309496423	2	0.85663084

*Note:* In the table, “Gene” denotes the gene name; “method” refers to the statistical test method (MR Egger and inverse variance weighted correspond to the MR‐Egger method and inverse variance weighting method, respectively); “*Q*” represents the heterogeneity test statistic; “*Q*_df” indicates the degrees of freedom; “*Q*_pval” denotes the *p*‐value for the heterogeneity test, used to determine whether heterogeneity exists among different genetic variants.

**TABLE 3:** The results of horizontal pleiotropy test.

Gene	egger_intercept	SE	*p*val
GPR65	0.001641553	0.014341583	0.919327596
LDLR	0.027525954	0.026057211	0.339145754
PLIN2	0.013692793	0.017437731	0.476214439
TGFBR3	0.016781662	0.028189199	0.612018244
TRIM14	0.014362911	0.028051215	0.69873821

*Note:* In the table, “Gene” denotes the gene name; “egger_intercept” represents the MR‐Egger intercept term used to assess horizontal pleiotropy; “SE” indicates the standard error; and “*p*val” signifies the *p*‐value used to determine whether horizontal pleiotropy is statistically significant.

**TABLE 4:** The results of Steiger test.

Gene	id.exposure	Direction	steiger_*p*val
PLIN2	eqtl‐a‐ENSG00000147872	True	5.67E‐208
GPR65	eqtl‐a‐ENSG00000140030	True	4.53E‐128
LDLR	eqtl‐a‐ENSG00000130164	True	1.60E‐54
TRIM14	eqtl‐a‐ENSG00000106785	True	3.45E‐73
TGFBR3	eqtl‐a‐ENSG00000069702	True	2.17E‐32

*Note:* In the table, “Gene” denotes the gene name; “id.exposure” represents the exposure factor identifier (eQTL‐associated number); “direction” indicates the causal direction determination (“True” indicates the causal direction aligns with expectations); “steiger_*p*val” is the *p*‐value from the Steiger test, used to verify whether genetic variation explains the exposure factor to a greater extent than the outcome, thereby confirming the validity of the causal direction.

In summary, LDLR, GPR65, TRIM14, PLIN2, and TGFBR3 were deemed as significant genes for further analysis.

### 3.3 Acquisition of the Four Biomarkers

**FIGURE 3:**
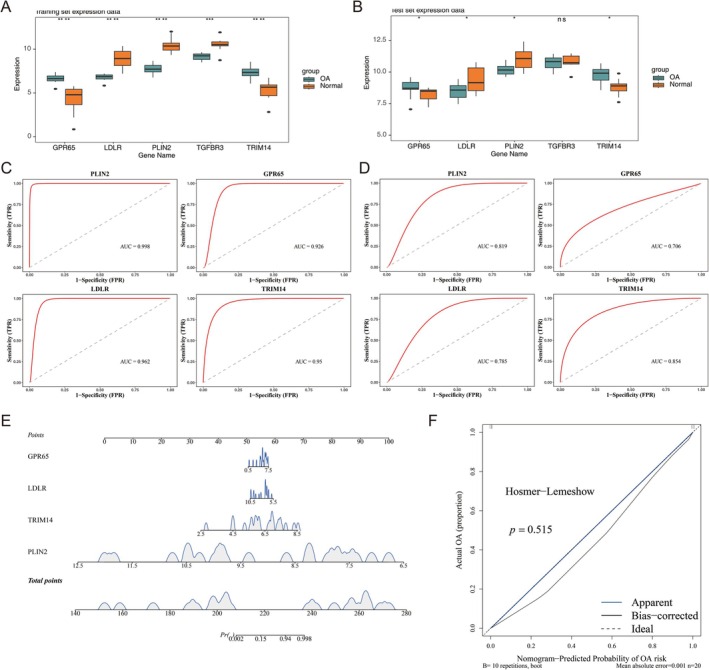
Acquisition of biomarkers. (A) Expression analysis of significant genes in the training set. The horizontal axis represents genes, and the vertical axis represents gene expression levels. Green: Osteoarthritis group; Orange: Normal group. ns: *p* > 0.05; *p < 0.05; ***p < 0.001; ****p < 0.0001. (B) Expression analysis of significant genes in the validation set. (C) ROC analysis of significant genes in the training set. The horizontal axis represents 1 − specificity (false positive rate, FPR), while the vertical axis represents sensitivity (true positive rate, TPR). The dashed line indicates a reference curve with no discriminative capability. The red curve represents the ROC curve for the key gene. The area under the curve (AUC) reflects the model's overall predictive accuracy, with a larger area indicating superior predictive performance. (D) ROC analysis of significant genes in the validation set. (E) Construction of Nomogram. (1) Left variable name: Each variable corresponds to a line marked with a scale, representing the variable's range of values, while the length of the line segment reflects the contribution of the factor to MI. (2) Score: Single item score represents the single item score corresponding to each variable at different values; The total score represents the sum of the individual scores corresponding to all variable values. (3) Prediction probability: Represents the risk of developing PD. (F) Calibration curve of Nomogram. Ideal represents the correction curve, the Apparel line represents the internal correction, and the BaPD corrected line represents the external correction curve of the model.

The expression analysis of the five significant genes revealed that LDLR and PLIN2 exhibited notably lower expression in OA samples, while GPR65 and TRIM14 showed notably higher expression across both the GSE55235 and GSE55457 datasets (*p* < 0.05) (Figure 3A,B). Further, ROC curve analysis indicated that the AUC values for GPR65, LDLR, PLIN2, and TRIM14 in both the GSE55235 and GSE55457 datasets were all greater than 0.7 (Figure 3C,D). Based on these results, GPR65, LDLR, PLIN2, and TRIM14 were deemed biomarkers for OA.

Subsequently, a risk model was constructed (Figure 3E), and the nomogram demonstrated that higher total points were associated with an increased risk of OA. The calibration curve further validated the accuracy of the model's predictions, with a *p* value of 0.515 (Figure 3F). Overall, these findings highlighted the strong efficacy of the nomogram in predicting OA, underscoring its potential as a valuable tool for clinical assessments.

### 3.4 Exploration Chromosomal Localization and Functions of Biomarkers

**FIGURE 4:**
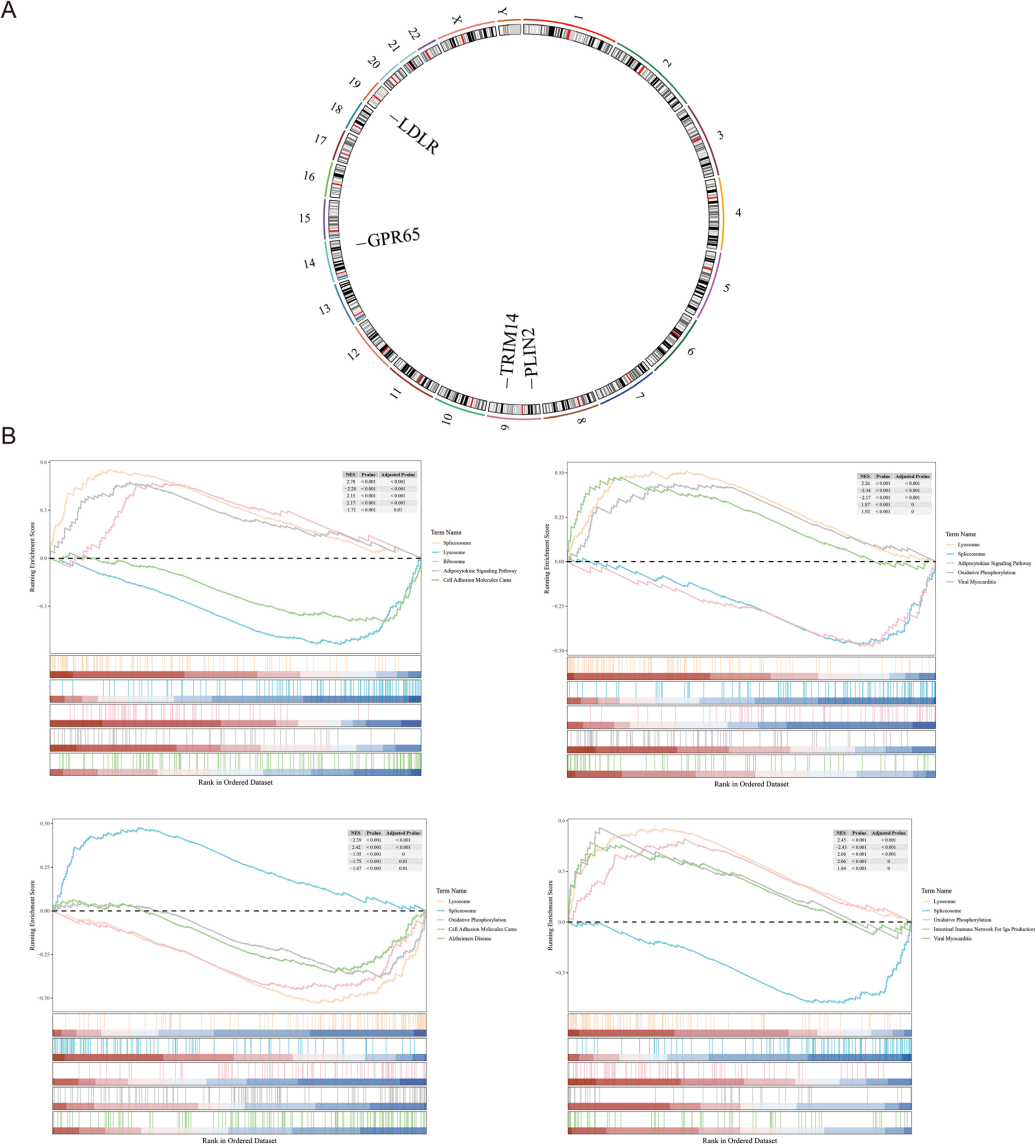
Chromosomal localization and functions of biomarkers. (A) Chromosome localization of key genes. The gene location is shown in the figure. (B) PLIN2 (top left), GPR65 (top right), LDLR (bottom left), TRIM14 (bottom right) GSEA enrichment analysis chart. Each image can be divided into two parts. Part 1: The top five lines are the line chart of gene enrichment score. The vertical axis represents the corresponding running ES, and there is a peak in the line graph, which is the enrichment score of this gene set. The genes before the peak are the core genes under this gene set. The horizontal axis represents each gene in this gene set, corresponding to the vertical line resembling a barcode in the second part; Part 2: The barcode‐like part is Hits, with each vertical line corresponding to a gene in the gene set.

Chromosomal localization analysis revealed that LDLR was located on chromosome 19, GPR65 was located on chromosome 14, and both TRIM14 and PLIN2 were located on chromosome 9 (Figure 4A). Additionally, GSEA showed that the four biomarkers were notably co‐enriched in pathways such as “lysosome” and “spliceosome,” which suggested these pathways play a key role in OA (Figure 4B). These findings suggested that disruptions in lysosomal function and spliceosomal machinery might affect the disease's progression, offering new avenues for targeted therapeutic strategies in OA management.

### 3.5 Exploration the Differences of Immune Infiltration in OA and Normal Samples

**FIGURE 5:**
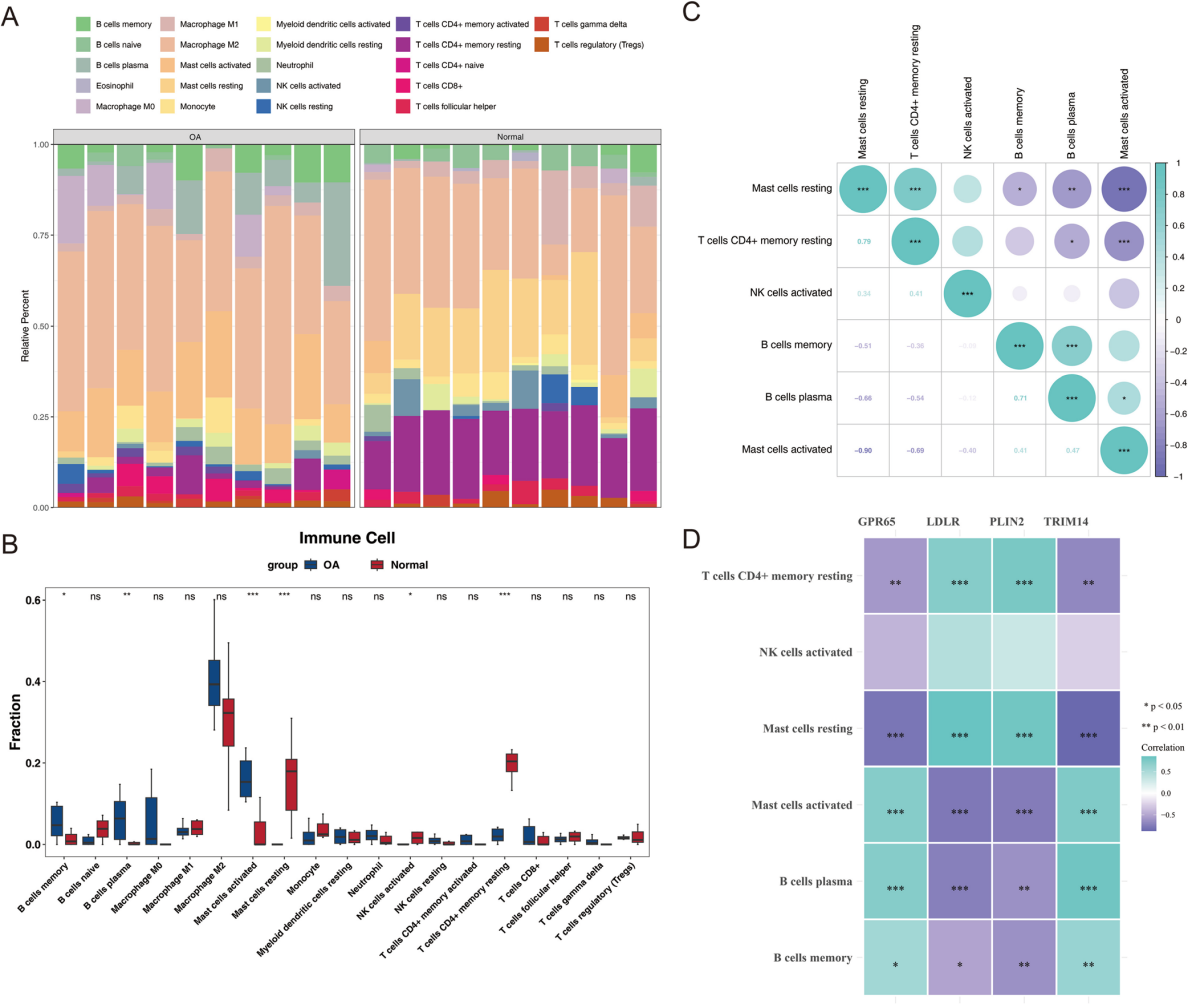
Immune infiltration analysis of biomarkers. (A) OA group versus control group immune infiltration abundance. Different colors represent different immune cells. Each column represents a sample, with the *y*‐axis showing the relative percentage of immune cell infiltration. (B) Differential immune cells. Blue: OA groups; Red: Normal groups. The horizontal axis represents immune cell types, while the vertical axis shows the infiltration ratio of immune cells across groups. ns: *p* > 0.05, *p < 0.05, **p < 0.01, ***p < 0.001. (C) Correlation between immune cells. The values represent correlation coefficients, with asterisks indicating significance. Light green denotes positive correlation, purple denotes negative correlation. *p < 0.05; **p < 0.01; ***p < 0.001. (D) The correlation between biomarkers and immune cells. Light green indicates positive correlation, purple indicates negative correlation, and darker colors indicate higher correlation. *p < 0.05; **p < 0.01; ***p < 0.001.

Figure 5A shows the immune infiltration profiles of 22 immune cell types in the samples from GSE55235 dataset. Obvious differences in immune cell infiltration were identified between OA and normal samples for six immune cell types (Figure 5B). For example, memory B cells showed higher infiltration levels in OA samples, whereas resting mast cells exhibited significantly lower infiltration levels in OA samples (*p* < 0.05). Further analysis revealed that resting mast cells had an obvious positive correlation with resting memory CD4+ T cells (cor = 0.79, *p* < 0.05) and an obvious negative correlation with activated mast cells (cor = −0.90, *p* < 0.05) (Figure 5C). Moreover, resting mast cells demonstrated the strongest positive correlation with LDLR (cor = 0.85, *p* < 0.01) and the most negative correlation with TRIM14 (cor = −0.87, *p* < 0.01), suggesting that resting mast cells might play a significant role in immune modulation in OA (Figure 5D; Table S7).

### 3.6 Revealing Potential Regulatory Mechanisms and Potential Therapeutic Drugs of OA

**FIGURE 6:**
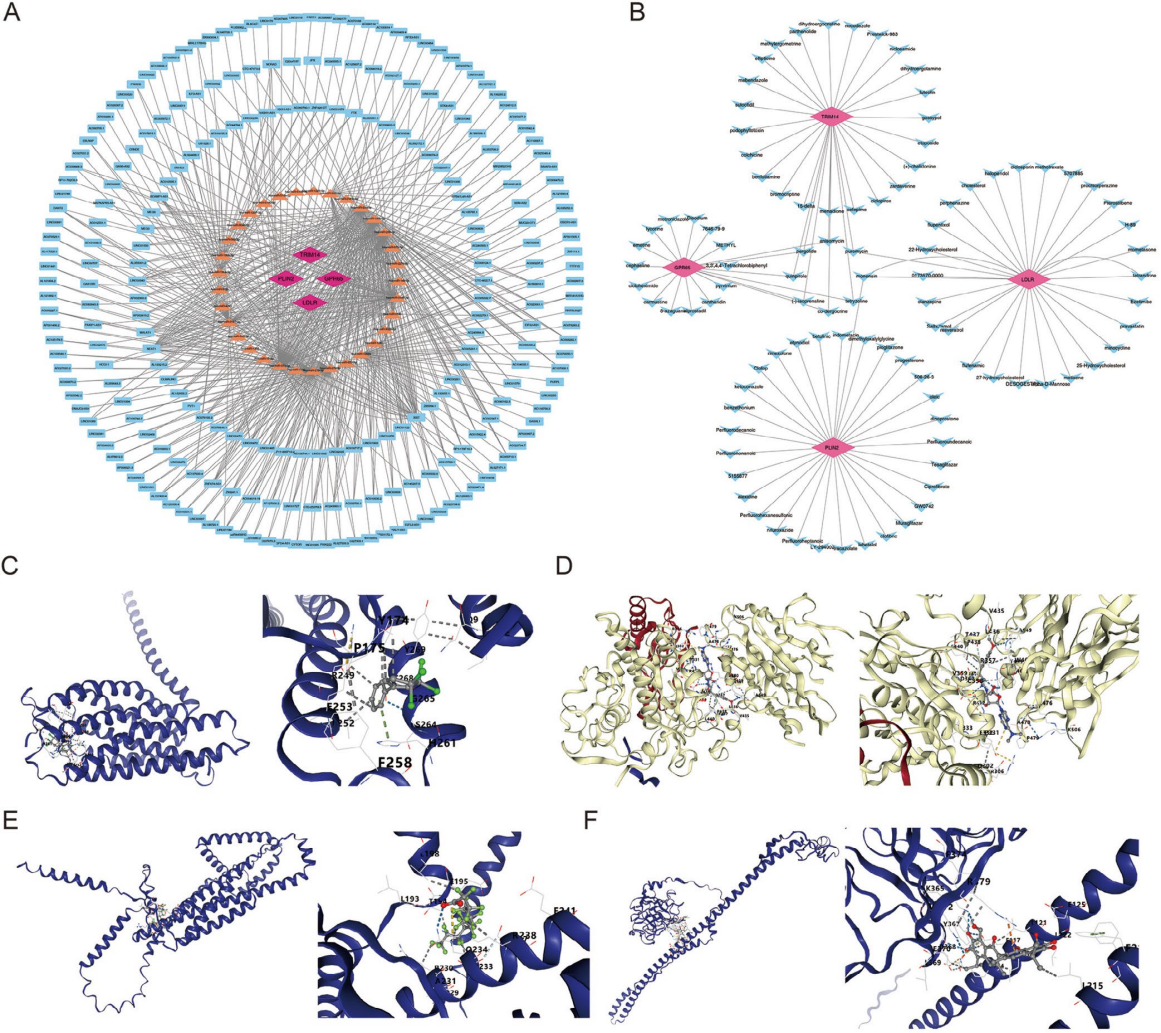
Potential regulatory mechanisms and drug prediction of biomarkers. (A) lncRNA–miRNA–mRNA network. The red diamonds indicate biomarkers, the yellow triangles represent miRNAs, the blue squares represent lncRNAs, and the connecting lines indicate interactions between them. (B) Drug prediction of biomarkers. Blue: Drug; Red: Biomarkers. (C–F) Molecular docking of biomarkers. (C) GPR65; (D) LDLR; (E) PLIN2; (F) TRIM14. The left panel shows the overall three‐dimensional structure of the protein encoded by the biomarker, with its spatial conformation depicted as a purple helical structure. The right panel presents a magnified view of the key binding region, highlighting the interactions between amino acid residues and the ligand. Dashed lines indicate hydrogen bonds or other intermolecular forces, while the green structure represents the bound ligand molecule. This visualization clearly illustrates the critical binding sites and interaction patterns between the protein and its ligand.

A total of 13 key miRNAs were predicted to target biomarkers, and 242 lncRNAs targeting these miRNAs were identified, leading to the construction of a lncRNA–miRNA–mRNA network. Notable relationships in this network included PLIN2‐hsa‐miR‐373‐3p‐EBLN3P, LDLR‐hsa‐miR‐27a‐3p‐XIST, GPR65‐hsa‐miR‐3146‐NEAT1, and TRIM14‐hsa‐miR‐223‐3p‐LINC00648, among others (Figure 6A). Furthermore, a set of 210, 111, 80, and 20 drugs was predicted to target LDLR, PLIN2, TRIM14, and GPR65, respectively. For example, anisomycin, pergolide, and quinpirole were found to co‐target TRIM14 and GPR65, while monensin co‐targeted LDLR and PLIN2 (Figure 6B). To further explore these interactions, molecular docking analysis was performed with four drugs: 3,3′,4,4′‐tetrachlorobiphenyl, puromycin, perfluoroundecanoic acid, and gossypol, identified to have the highest interaction potential with GPR65, LDLR, PLIN2, and TRIM14. The results of the molecular docking revealed that GPR65 and 3,3′,4,4′‐tetrachlorobiphenyl exhibited a favorable binding energy of −7.6 kcal/mol, with key interactions involving residues P175 and H261 (Figure 6C). LDLR and puromycin demonstrated a binding energy of −9.3 kcal/mol, with critical interactions at residues K506 and R357 (Figure 6D). PLIN2 and perfluoroundecanoic acid showed a binding energy of −7.1 kcal/mol, with key interactions at residues L193 and A231 (Figure 6E). Lastly, TRIM14 and gossypol demonstrated a binding energy of −7.6 kcal/mol, with critical interactions at residues K365 and Y367 (Figure 6F).

### 3.7 Identification T Cells and Mast Cells as Key Cells for OA

**FIGURE 7:**
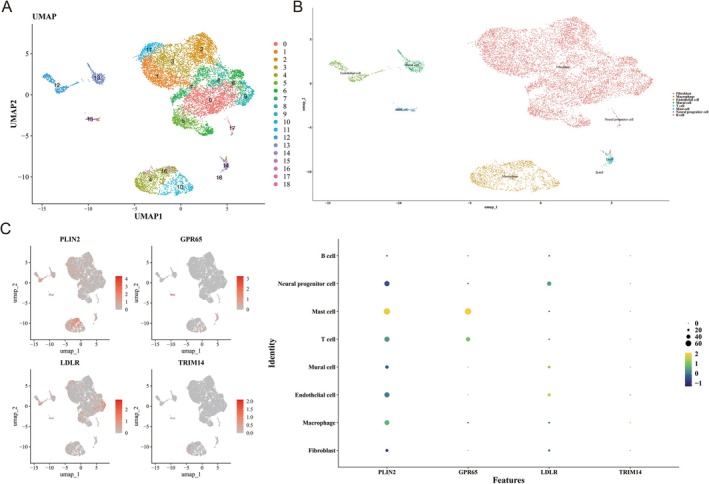
Acquisition of key cell. (A) UMAP diagram before annotation. The horizontal axis represents UMAP1, and the vertical axis represents UMAP2. Different colors denote distinct cell clusters (labeled 0–18), with each point representing a single cell. (B) UMAP diagram after annotation. Different colors represent different cells. (C) UMAP plot displays the distribution of biomarkers expression in cells. The gray bottom represents the distribution of cells, while the red indicates expression in this area. Bubble plot of biomarker expression in cells. The vertical axis represents cell type (Identity), while the horizontal axis represents biomarkers (Features). Point color indicates gene expression levels, and point size reflects the number of cells expressing that biomarker.

Initially, 10 248 cells and 20 490 genes were retained after filtering out ineligible cells and genes (Figure S6A). A set of 2000 HVGs was identified, with the top 10 HVGs labeled (Figure S6B). PCA was performed, selecting the top 30 PCs for further analysis (*p* < 0.05) (Figure S6C). Using UMAP, the cells were categorized into 19 distinct clusters (Figure 7A). These clusters were then annotated as B cells, neural progenitor cells, mast cells, T cells, mural cells, endothelial cells, macrophages, and fibroblasts (Figure 7B; Table S8). Notably, PLIN2 and GPR65 were highly expressed in mast cells and T cells (Figure 7C), suggesting that these two cell types are key players in OA. This analysis identified and characterized 19 distinct cell clusters, with mast cells and T cells showing high expression of PLIN2 and GPR65, highlighting their potential role as key cells in OA.

### 3.8 Exploration Interactions and Differentiation Trajectories of Key Cells

**FIGURE 8:**
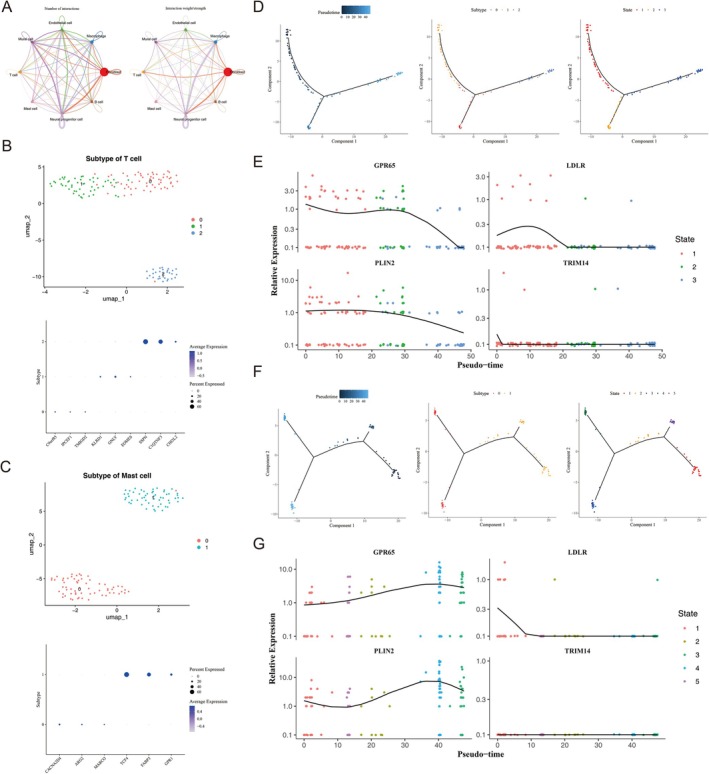
Interactions and differentiation trajectories of key cells. (A) Number of cell–cell interactions (left) and weighted (right) for all annotated cell clusters. Different colors represent different cell types, and the thickness of lines represents the strength of cell interactions. The thicker the lines, the stronger the cell interactions. The size of the circle represents the number of cells. (B) UMAP diagram of T cell re clustering (top) and bubble plot of annotated genes (bottom). Each point in the UMAP plot represents a cell, with colors denoting different clustering groups. In the bubble plot, the *x*‐axis shows marker genes, the *y*‐axis displays cluster IDs, bubble size indicates expression levels, and bubble color reflects average expression levels. (C) UMAP diagram of mast cell re clustering (top) and bubble plot of annotated genes (bottom). Different colors represent different cell subtypes. The larger the dot, the greater the expression. (D) Differentiation trajectory diagram of T cell pseudotime analysis. Dark blue represents early differentiation, light blue represents late differentiation (left). Cell proportion of three T cell subtypes along the differentiation trajectory (middle). T cell differentiation comprises three stages, with state 1 being the earliest differentiation stage. (E) Dynamic trend diagram of relative biomarker expression levels along the T cell differentiation trajectory, plotted against pseudotime and cell state. The *x*‐axis represents pseudotime (0–50 indicates developmental progression), the *y*‐axis represents relative expression levels, and the curves depict the relative expression trends of biomarkers. Different colors represent cell states (types 1, 2, 3), illustrating the temporal and state‐dependent expression pattern changes of the GPR65 gene during T cell development. Scatter points represent individual T cells, with colors corresponding to different differentiation states. (F) Differentiation trajectory diagram of mast cells. (G) Dynamic trend diagram of relative expression levels of biomarkers in mast cell differentiation trajectories as a function of pseudotime and cell state.

Cell‐to‐cell communication analysis revealed frequent interactions between neural progenitor cells and various other cell types, including fibroblasts, mural cells, T cells, and others (Figure 8A). These findings suggested that neural progenitor cells might play a central role in mediating cell signaling and communication within the tissue microenvironment. Additionally, we performed dimensionality reduction and clustering analysis on the key cells, which revealed that T cells could be divided into three subtypes, while mast cells were grouped into two subtypes (Figure 8B,C). Pseudo‐temporal analysis of T cell differentiation revealed a progressive trajectory from left (dark blue) to right (light blue), with distinct classification into three subtypes and three developmental states (Figure 8D). State 1 represented the earliest stage of differentiation. During this process, the expression of the biomarker GPR65 initially declined in the early stage, increased in the middle stage, and then rapidly declined in the late stage. LDLR expression increased in State 1 before decreasing, with no significant changes observed in States 2 and 3. PLIN2 expression steadily decreased throughout the differentiation process, while TRIM14 expression remained unchanged over time (Figure 8E). Additionally, the differentiation trajectory of mast cells progressed in the opposite direction, from right (dark blue) to left (light blue), with cells classified into two subtypes and five distinct states (Figure 8F). In mast cells, the overall expression trend of GPR65 and PLIN2 was an increase over time. LDLR expression was observed only in State 1, where it showed a declining trend. TRIM14 expression did not show significant changes during mast cell differentiation (Figure 8G). Overall, the differentiation trajectories of both T cells and mast cells reveal temporal changes in the expression of four biomarkers. These changes likely reflect important biological processes that contribute to disease progression.

## 4 Discussion

Osteoarthritis OA is recognized as a chronic inflammatory progressive disease [3, 15]. Current research primarily focuses on the interactions among inflammatory factors, immune cells, and joint tissues [3, 57], while the potential mechanisms of TEX in OA remain underreported. This study, leveraging data from the GEO database, identified four T cell exhaustion‐related genes (GPR65, LDLR, PLIN2, TRIM14) in OA through a series of analyses, including differential expression analysis, MR analysis, gene expression analysis, and ROC analysis. Further enrichment analysis, immune infiltration analysis, and drug prediction analysis were conducted to explore the potential mechanisms of these genes in OA patients.

Perilipin 2 (PLIN2) is a lipid droplet‐associated protein primarily involved in the storage, formation, and stabilization of lipid droplets [58]. Currently, the aspects of the regulation of lipid metabolism by PLIN2 include mediating lipid hydrolysis and participating in the regulation of lipid recombination, while the involved mechanisms include phospholipid homeostasis and mitochondrial function [59]. Previous studies have indicated a significant correlation between PLIN2 and osteoarthritis (OA), although its specific mechanisms remain poorly understood [60]. Based on the IVW results of MR in this study, PLIN2 may serve as a potential protective factor against OA, providing important insights for further exploration of its molecular mechanisms. The low‐density lipoprotein receptor (LDLR), encoded by the LDLR gene on chromosome 19, is a 45 kb protein comprising 839 amino acids [61]. Its well‐established physiological function involves the internalization of lipoproteins, primarily low‐density lipoprotein (LDL), thereby directly regulating blood lipid levels [62]. Earlier research has indicated that LDL accumulation during experimental OA contributes to osteophyte formation and ectopic bone growth, a process associated with the activation of synovial macrophages [63]. Additionally, MR study focusing on genetic lipid‐lowering targets and OA has established a connection between LDLR and the development of both hip and knee osteoarthritis [64]. These evidence complements the results defined by MR in this study, indicating that LDLR has a potential effect on OA.

Given that both LDLR and PLIN2 are associated with lipid metabolism, this indicates that there may be a certain relationship between lipid metabolism and osteoarthritis. Imbalanced lipid metabolism pathways can generate pro‐inflammatory substances, which directly impact OA development [65]. Research shows. Downregulation of LDLR expression can directly lead to T cell receptor (TCR) signaling dysfunction, promoting abnormal upregulation of depletion markers such as PD‐1 and TIM‐3, while reducing the secretion of effector factors such as IFN‐γ and TNF‐α, exacerbating T cell metabolic defects [66]. Downregulation of PPLIN2 synergizes with LDLR deficiency, leading to impaired energy metabolism by disrupting mitochondrial function, triggering accumulation of reactive oxygen species (ROS), and oxidative stress [67]. Oxidative stress further activates the NF‐κB pathway, inducing the release of pro‐inflammatory cytokines such as TNF‐α and IL‐6, ultimately exacerbating T cell dysfunction [68, 69]. T cell exhaustion not only weakens the immune response, but also directly limits the repair ability of regulatory T cells (Tregs), leading to hindered joint tissue regeneration and accelerating the destruction of OA cartilage and bone structures [70]. Overexpression of inflammatory factors such as IL‐1β, IL‐8, and TNF‐α has been observed in the synovial membrane of OA patients [64]. A pharmacological study reported and confirmed that statins have a mechanism to reduce the levels of inflammatory factors such as IL‐1β [71]. Another large retrospective cohort research has provided more robust evidence, demonstrating that statins can reduce the progression rate of knee osteoarthritis by 50% [72]. Therefore, lipid metabolism may indirectly contribute to the pathology of OA through inflammation, and lipid‐lowering drugs can improve the situation to a certain extent. Targeting LDLR to ameliorate OA is theoretically a promising direction. In addition, abnormal adipokines and lipid deposition are important components of the pathological mechanism of OA [73]. Although reducing PLIN2 expression has been shown to improve hepatic lipid deposition in mice [74]. However, it is not yet clear whether PLIN2 affects disease progression by regulating lipid deposition in OA joint tissues, which provides an important research direction for exploring PLIN2 as an intervention target for OA metabolism. In summary, LDLR and PLIN2 regulate lipid metabolism and affect T cell exhaustion, thereby participating in the progression of OA. They are not only key molecules connecting lipid metabolism and TEX, but also provide theoretical basis and research entry points for metabolic targeted therapy of OA.

G protein‐coupled receptor 65 (GPR65) is a type of G protein coupled receptor primarily expressed on immune cells, including T cells, macrophages, and dendritic cells [75]. It plays an important role in regulating T cell function. Research has shown that GPR65 can promote the pathogenicity of Th17 cells and induce T cell apoptosis [76]. This discovery reveals the potential role of GPR65 in TEX, suggesting that it may indirectly participate in the pathogenesis of OA by affecting T cell function. Its physiological function is mainly reflected in regulating the immune response and acid–base balance through cellular signaling pathways [77]. In the inflammatory microenvironment, the expression level of GPR65 is significantly upregulated [78]. It further amplifies its impact on immune cell function. A recent study has found that GPR65 promotes joint pain by activating fibroblast‐like synoviocytes and releasing pro‐inflammatory mediators in both animal models and human OA patients [79]. The potential mechanism involved may be related to GPR65 increasing pain sensation induced by mechanical stimuli through the modulation of inflammatory mediators and neuronal sensitization [80]. In this study, MR identified GPR65 as a risk factor for OA, which is consistent with current evidence. In addition, in the chronic inflammatory environment of OA, the acidosis phenomenon or low pH value that occurs locally in the joints can further activate GPR65 [79]. The activated GPR65 triggers downstream signal transduction processes, which then contribute to the progression of inflammation and pain in OA. In summary, GPR65 can be considered a key target associated with OA‐related inflammation and pain. It may participate in immune disorders by regulating T cell apoptosis and functional imbalance, and exacerbate joint inflammation and pain by responding to changes in the local microenvironment of OA. It has become a key molecule connecting TEX and OA pathological processes, providing a new perspective for understanding the immune metabolism mechanism of OA. However, its specific regulatory network still needs further research to verify.

TRIM14, an intracellular E3 ubiquitin ligase, plays a critical role in antiviral defense, immune responses and inflammation regulation [81]. It is worth noting that TRIM14 can significantly promote the exhaustion of CD8+ T cells by activating the IFN‐β signaling pathway. This characteristic has been confirmed in the tumor microenvironment, and its high expression can inhibit the anti‐tumor activity of T cells, even leading to resistance to PD‐1/PD‐L1 immunotherapy [82]. It indicates a close association with TEX. In the context of OA, TRIM14 expression is closely associated with innate immune responses, particularly through its regulation of the IFN‐β and NF‐κB signaling pathways, which drive inflammation and joint degeneration [83]. Additionally, TRIM14 influences chronic inflammatory pain in OA patients and OA rats by interacting with the IκBα signaling pathway [84]. Existing research has confirmed that inhibiting or downregulating TRIM14 can effectively alleviate the progression of OA [85]. This provides strong support for identifying it as a risk factor for OA in this study. In summary, TRIM14 may become an important molecule connecting the pathological mechanisms of TEX and OA by linking the inflammatory and degenerative processes of TEX and OA. Further research on the molecular mechanism of TRIM14 in OA can provide a deeper understanding of its potential as a diagnostic biomarker and therapeutic intervention.

ROC curve analysis validated the diagnostic value of GPR65, LDLR, PLIN2, and TRIM14 as biomarkers for OA. The high expression of GPR65 and TRIM14 may serve as early warning signals for OA, while the low expression of LDLR and PLIN2 likely reflects pathological changes in OA. The results of the nomogram indicate that the risk model developed in this study provides clinicians with a simple and effective tool for assessing OA risk. Clinicians can quickly quantify the risk of OA onset or the degree of disease progression by detecting the expression levels of the four targets, combined with basic indicators such as age and BMI; this method is particularly suitable for OA risk screening in primary medical institutions. From a longer‐term therapeutic translation perspective, these four targets can serve as potential therapeutic targets for different intervention stages of OA, specifically improving the local joint metabolic and inflammatory microenvironment and delaying the progression of cartilage degeneration. However, the diagnostic efficacy of these biomarkers has only been verified in the synovial tissue dataset. Further verification is still needed in serum samples, as well as in multicenter, large‐sample clinical cohort studies, to confirm their diagnostic efficacy across different regions and populations, and to clarify the reference thresholds for their use as diagnostic biomarkers. Additionally, it is necessary to combine indicators such as age and BMI to quickly quantify the risk of OA onset or the degree of disease progression.

GSEA analysis revealed that four biomarkers were co‐enriched in pathways such as the spliceosome and lysosome. The lysosome serves as the primary degradation and recycling center within cells, maintaining intracellular homeostasis by breaking down organelles and degrading abnormal macromolecules [86]. Destabilization of the lysosome can activate inflammasomes, causing chondrocyte pyroptosis and promoting pathological hydroxyapatite deposition in OA cartilage [87]. Moreover, dysfunction of the lysosome disrupts autophagy, leading to inflammatory cell death in chondrocytes and further aggravating the progression of OA [88]. The spliceosome is a highly dynamic RNA‐protein complex composed of five small nuclear ribonucleoprotein particles and numerous auxiliary proteins, which plays a critical role in the precise regulation of gene expression [89]. Research has revealed that small nucleolar RNAs fine‐tune the function of the spliceosome, thereby controlling the splicing and expression of genes associated with OA and impacting the phenotype and function of chondrocytes [90]. Based on the above evidence, the four key targets defined in this study may participate in the pathological mechanisms of OA by acting on the spliceosome or lysosome.

Through single‐cell analysis, this study emphasizes the importance of mast cells and T cells in OA. In OA, existing evidence indicates that the activation of mast cells contributes to OA pathogenesis through multiple mechanisms, including inflammatory factor infiltration, cartilage degradation, and the induction of chondrocyte apoptosis [91]. As mentioned before, the release of inflammatory factors contributes to the persistent exacerbation of intra‐articular inflammation. Additionally, cartilage breakdown and cell death cause thinning and cracks, leading to pain and loss of function [92]. It has been clearly demonstrated that GPR65 is expressed in mast cells [93], and mast cells participate in lipid metabolism related to LDLR [94]. Mast cells involved in lipid droplet formation and stabilization are also related to PLIN2 [95]. Therefore, mast cells may play a critical role in the pathological mechanisms of OA, potentially interacting with GPR65, LDLR, PLIN2, or TRIM14 to mediate inflammatory responses and cartilage degradation processes. Notably, the infiltration of T cells into the joint is regarded as a specific hallmark of OA [96]. Moreover, the activation and differentiation of T cells, along with their production of inflammatory factors, directly contribute to the pathological progression of OA [97]. Collectively, these findings underscore the central role of T cells and mast cells in the pathogenesis of OA. Understanding how these immune cells interact and are regulated in OA will help develop better treatments.

This study identified four TEX‐related biomarkers in OA, namely GPR65, LDLR, PLIN2, and TRIM14. These findings preliminarily provide valuable insights into the research on T cell exhaustion in OA. However, this study still has certain limitations. The current research is only based on bioinformatics analysis of public databases, lacking validation of the correlation between gender and clinical routine indicators (such as joint fluid inflammatory factor levels, imaging grading, etc.), and the expression of the biomarkers also lacks verification in an independent clinical cohort. Then, the specific molecular mechanisms by which these four biomarkers regulate T cell exhaustion, lysosomal function, and spliceosome pathways also lack experimental validation. The relevant network analysis and molecular docking results are only at the predictive level and have not directly confirmed their therapeutic potential. Therefore, in the future, we plan to include an independent clinical sample cohort to validate the expression of the biomarkers in OA patients and healthy individuals via qRT‐PCR/immunohistochemistry, further verify the specific mechanism of action between selected genes and T cell exhaustion through experiments, and use cell experiments to test their roles in lysosomal function and splicing regulation, as well as the interaction between non coding RNA and marker mRNA. Secondly, using animal models to comprehensively evaluate the expression characteristics of these biomarkers and their functions in the OA process. In addition, in order to better understand the molecular mechanism of OA, we plan to verify the regulatory effect of drugs on biomarker function and their improvement effect on OA phenotype through experiments in the future. At the same time, it is planned to include a larger sample of multi omics data to validate the stability of the regulatory network through gender stratification analysis, and to combine functional experiments to verify the interaction between TEXRGs and conventional clinical indicators, comprehensively evaluating the localization of TEXRGs in OA pathology.

## 5 Conclusion

This study identified GPR65, LDLR, PLIN2, and TRIM14 as biomarkers associated with T cell depletion in osteoarthritis, which may serve as potential targets for OA treatment and warrant further investigation.

## Author Contributions

Kun Zhang and Lun Wan conceived and designed the research; Jiahong Li and Yan Zhang performed the research and acquired the data; Wei Fang, Fei Lan, Qian Yan, Jiang Hu, and Chengwei Xiao analyzed and interpreted the data. All authors were involved in drafting and revising the manuscript.

## Funding

The authors have nothing to report.

## Disclosure

Declaration of AI and AI‐assisted technologies: There was no use of AI and AI‐assisted technologies in scientific writing.

## Ethics Statement

The authors have nothing to report.

## Consent

The authors have nothing to report.

## Conflicts of Interest

The authors declare no conflicts of interest.

## Supporting information


**Data S1:** fsb271483‐sup‐0001‐DataS1.zip.

## Data Availability

The data that support the findings of this study are openly available in the Gene Expression Omnibus (GEO) database at https://www.ncbi.nlm.nih.gov/geo/, GSE55235, GSE55457, GSE152805 and IEU Open GWAS database at https://gwas.mrcieu.ac.uk/, ebi‐a‐GCST005814.
