# Supplementary material for: Mining Potential Therapeutic Targets for T Cell Exhaustion in Osteoarthritis by Integrating Mendelian Randomization and Single‐Cell Sequencing
Source: FASEB J. 2026 Jan 28;40(2):e71483. doi: 10.1096/fj.202503295R (PMC12850000; doi:10.1096/fj.202503295R)
Supplement: Supplementary file 1 — Data S1: fsb271483‐sup‐0001‐DataS1.zip. [file FSB2-40-e71483-s001.zip › 202503295R-sup-0017-SI_Text-S01.docx]

Supplementary Materials

Supplementary Figure S1 Verification of MR analysis results. (A) MR analysis of 5 genes tested using IVW and EMIC methods. (B) Q-Q plot of MR analysis of 5 genes.

Supplementary Figure S2 MR analysis of candidate genes. (A) Scatter plot of correlation analysis between exposure factors and outcomes. The colored lines represent the fitting results of different MR algorithms. A regular slope of the line indicates a risk factor, while a negative slope indicates a safety factor. When there is an intercept, it implies the presence of confounding factors. (B) Forest plot of instrumental variable outcome effect estimation. The solid line is completely to the left of 0, indicating that the result estimated by this SNP is that an increase in exposure factors can reduce the risk of outcome variables; The solid line is completely to the right of 0, indicating that the result estimated by this SNP is that an increase in exposure factors can increase the risk of outcome variables. (C) The funnel plot of MR randomness judgment. (D) LOO's Forest Map. The blue line represents the overall effect value, the red dot represents the IVW estimate after removing the SNP, and the blue dot represents the IVW estimate for all SNPs. If the horizontal deviation of the red dot from the blue dot is relatively close, it indicates that the MR result can withstand the sensitivity analysis test

Supplementary Figure S3 Identify of key cells. (A) Before single-cell data quality control (top), after B single-cell data quality control (bottom). (B) Screening of highly variable genes. The red dots represent the first 2000 highly variable genes. (C) PCA dimensionality reduction (top left) scree plot (top right) B Jackstrand plot (bottom).

Supplementary Table S1 Summary table of genes involved in TEX.

Supplementary Table S2 DEGs.

Supplementary Table S3 Summary table of GO and KEGG enrichment analysis of candidate genes.

Supplementary Table S4 Summary table of MR analysis of candidate genes.

Supplementary Table S5 MR-PRESSO analysis results for causal genes associated with OA.

Supplementary Table S6 Global Test Results for Horizontal Pleiotropy of Causal Genes Linked to OA.

Supplementary Table S7 Correlation between differential immune cells and biomarkers.

Supplementary Table S8 The information of marker genes.
