# Supplementary material for: Mining Potential Therapeutic Targets for T Cell Exhaustion in Osteoarthritis by Integrating Mendelian Randomization and Single‐Cell Sequencing
Source: FASEB J. 2026 Jan 28;40(2):e71483. doi: 10.1096/fj.202503295R (PMC12850000; doi:10.1096/fj.202503295R)
Supplement: Supplementary file 1 — Data S1: fsb271483‐sup‐0001‐DataS1.zip. [file FSB2-40-e71483-s001.zip › fsb271483-sup-0012-TableS6.docx]

| Cluster | Cell | Maker gene | | |
| --- | --- | --- | --- | --- |
| 0 | Fibroblast | COL3A1 | COL5A1 |  |
| 1 | Fibroblast | FBLN2 | MMP2 |  |
| 2 | Fibroblast | IGFBP5 | MT1A | SPARCL1 |
| 3 | Fibroblast | C1R | COL14A1 | CXCL12 |
| 4 | Macrophage | CD163 | C1QA |  |
| 5 | Fibroblast | COL14A1 | PTGDS |  |
| 6 | Fibroblast | TNFAIP6 | CD55 |  |
| 7 | Fibroblast | CTGF | CD55 |  |
| 8 | Fibroblast | BMP2 | FGF2 |  |
| 9 | Fibroblast | FGF2 | COL2A1 | BMP2 |
| 10 | Macrophage | CLEC5A | CCL22 | BCL2A1 |
| 11 | Fibroblast | IL6 | GGT5 | APOD |
| 12 | Endothelial cell | ABCB1 | CALCRL | VWF |
| 13 | Mural cell | ACTA2 | MCAM |  |
| 14 | T cell | CD2 | CD3D |  |
| 15 | Mast cell | HDC | CTSG | CALB2 |
| 16 | Macrophage | CD163 | C1QA | TNF |
| 17 | Neural progenitor cell | BIRC5 | CCNA2 | AURKB |
| 18 | B cell | BLNK | AIM2 |  |

**Supplementary Table S8 The information of marker genes**
