# Supplementary figures and images for: Mining Potential Therapeutic Targets for T Cell Exhaustion in Osteoarthritis by Integrating Mendelian Randomization and Single‐Cell Sequencing
Source: FASEB J. 2026 Jan 28;40(2):e71483. doi: 10.1096/fj.202503295R (PMC12850000; doi:10.1096/fj.202503295R)

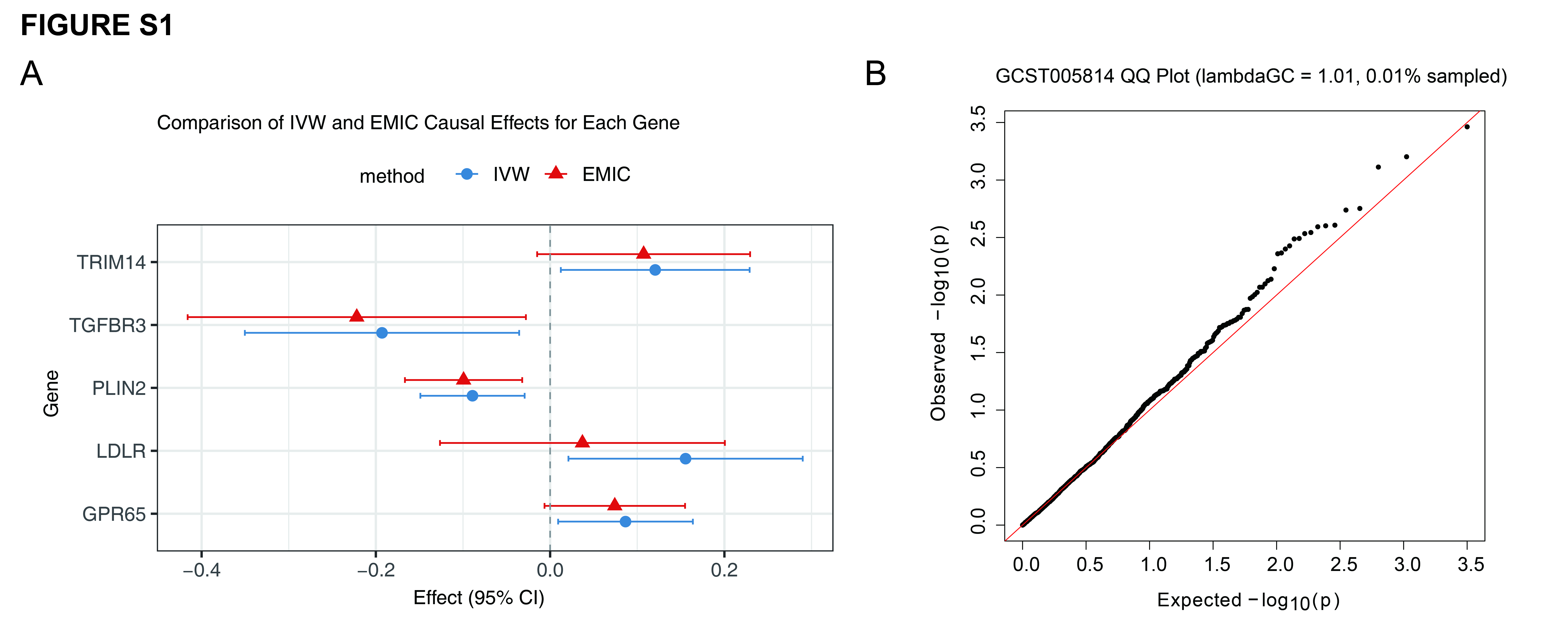

Supplement: Supplementary file 1 — Data S1: fsb271483‐sup‐0001‐DataS1.zip. [file FSB2-40-e71483-s001.zip › fsb271483-sup-0001-FigureS1.tif]
